# Quality Characteristics of Rice-Based Ice Creams with Different Amylose Contents

**DOI:** 10.3390/foods12071518

**Published:** 2023-04-03

**Authors:** Gi-Un Seong, Ji-Yoon Kim, Jung-Soo Kim, Sae-Ul Jeong, Jun-Hyeon Cho, Ji-Yoon Lee, Sais-Beul Lee, Nkulu-Rolly Kabange, Dong-Soo Park, Kwang-Deog Moon, Ju-Won Kang

**Affiliations:** 1 Department of Southern Area Crop Science, National Institute of Crop Science, Rural Development Administration, Miryang 50424, Republic of Korea; 2 School of Food Science and Technology, Kyungpook National University, Daegu 41566, Republic of Korea; 3 National Institute of Crop Science, Rural Development Administration, Sangju 37139, Republic of Korea

**Keywords:** rice, ice cream, amylose content, physicochemical properties, sensory quality

## Abstract

Ice cream consumption has increased over the years. In this study, we investigated the potential of using rice varieties with varying amylose contents for ice cream production. We analyzed the physical and chemical properties and sensory quality characteristics (appearance, taste, texture, chewiness, aroma, and rice flavor) of rice-based ice cream made from five varieties with low and high amylose levels. To make the ice cream, we ground rice into a fine powder and combined it with skim milk powder, butter, sugar, glycerin esters of fatty acids, locust bean gum, and water to form a gelatinized mixture. This mixture was then aged, frozen, and hardened. The ice cream’s key quality characteristics, such as viscosity (2170–25,030 cP), hardness (4.27–49.55 N cm^−2^), and overrun (17.95–46.99%), showed a wide range. Ice cream made from Saemimyeon (high amylose content rice variety) exhibited the highest hardness value (49.55 N cm^−2^) among the varieties tested, but had relatively low viscosity (4030 cP), overrun (17.95%), and drip-through (0.75 g/min) values. These findings suggest that rice varieties with different amylose contents are suitable for making ice cream and have the potential to expand the rice processing market and increase its value.

## 1. Introduction

In the context of global warming, mainly caused by the persistent emission of greenhouse gases (GHGs) that exacerbate climate change, agriculture is regarded as both a source and sink of atmospheric GHGs. Agriculture (crop production, land use, forest, and livestock) accounts for about 18% of global GHG emissions, with livestock and rice cultivation contributing about 6% and 1%, respectively [1,2].

Rice (*Orzya sativa* L.) is a major source of energy, consumed as a staple food by more than half of the world’s population, especially in Asia [3]. Rice varieties are categorized based on their amylose content, ranging from 0% to >24%, and low amylose rice offers high viscosity and swelling properties, while high amylose rice has a high non-digestible starch content [4,5] Differences in amylose content result in variation in the granular structure, physicochemical properties, and product quality [6].

The amylose content of rice is an important factor that impacts its cooking, processing, and eating qualities [7] Amylose content is controlled by various genes, and granule-bound starch synthase (GBSS) encoded by the waxy (wx) gene plays a critical role in amylose synthesis in plants [8,9]. In semi-waxy rice, the amylose content usually ranges from 6–15%, and genes such as wx-mq, du1 and du12 have been identified to affect amylose content (Figure S1) [10,11,12]. Functional alleles wx^a^ and wx^b^ are mainly found in *Oryza sativa* ssp. indica and *Oryza sativa* ssp. japonica, respectively [13,14]. Additionally, the simultaneous insertion of BGSS1 and SBE3 can increase amylose content up to 40%, as well as the content of resistant starch, a type of undigested starch [15]. With the advancement of molecular breeding technologies, genetic markers can now be used to more efficiently select desirable traits in breeding programs.

In the Republic of Korea, per capita domestic rice consumption has decreased by 20.1% over the last 10 years, driven by factors such as the Westernization of the diet and an increase in dual-income couples. To promote rice consumption, researchers are studying products such as rice cookies, rice noodles, rice beverages, and ready-to-eat rice [16,17,18]. Developing rice varieties suitable for processing is also a focus of research [19], but processed rice consumption accounts for only 6% of domestic production [20]. Therefore, there is a need for industrialization and research on processed rice food products to adapt to changing social and economic conditions, such as an aging population and consumer diversification (Tables S1–S3).

Ice cream is a popular frozen dessert enjoyed by people of all ages and sexes worldwide [21]. It is made by blending milk, fat, sweeteners, stabilizers, emulsifiers, and other additives [22]. The texture and palatability of ice cream are also impacted by factors such as additives, formulations, and processing methods [23]. Frozen products such as ice cream offer the opportunity to add nutritional or functional value due to low storage temperature and ingredient stabilization, making them an attractive option to deliver additional benefits beyond basic nutrition [24]. There is a growing trend toward research focused on creating healthy and high-value ice cream for consumers [25,26,27]. For years, dairy products have been the major component in the ice cream making process, while cereal products, such rice grains, have not received the desired attention, yet they have interesting nutraceutical properties and health benefits for many people worldwide [28]. Researchers are exploring the possibility of substituting or reducing the use of dairy products in ice cream making for several reasons, including fat content, flavor, and environmental aspects [29]. While there is ample research on creating low-fat, low-calorie ice cream products by adding health-promoting ingredients, limited research has been conducted on rice-based ice cream [30,31].

This study aimed at assessing rice varieties having differential physiochemical properties and quality characteristics for rice-based ice cream making, considering that rice offers good nutraceutical properties and is widely cultivated globally. Therefore, a group of rice varieties with different amylose contents, widely cultivated in the Republic of Korea, was used. The results identified rice cultivars with desirable physiochemical properties that can be used as sources of raw material for rice-based ice cream making.

## 2. Materials and Methods

### 2.1. Materials

The rice (*Oryza sativa* L.) varieties used in the experiments were Baegokchal [32], Miho [33], Saeilmi [34], Saemimyeon [19], and Dodamssal [35], with five different amylose contents. Among these varieties, Baegokchal, Miho, Sailmi, and Dodamssal are indeed japonica types, whereas Saemimyeon is a tongil-type rice [36]. The rice varieties used were representatively cultivated in South Korea, with samples certified by the Rural Development Administration for manufacturing. All of the rice grains used were cultivated in the experimental field (altitude: 11 m 35°29′31.4″ N, 128°44′31.4″ E), located in the Rural Development Administration (Miryang, Republic of Korea) in 2020. Dried rough rice samples were husked using an impeller husker (FC2K, Yamamoto, Yamagata, Japan) until their weight was 90% that of the starting brown rice sample. Then, milling was performed using a blade mill (Korea Powder System Co., Ltd., Incheon, Republic of Korea) with a built-in 180 mesh standard sieve. Rice flour was stored in a 4 °C refrigerator until use. Skim milk powder (Lactokorea Co., Cheongyang, Republic of Korea), butter (Anchor Food Professionals Co., Ltd., Fremantle, Australia), white sugar (CJ Cheiljedang Co., Ltd., Seoul, Republic of Korea), locust bean gum (ES Food Cor., Gunpo, Republic of Korea), and mono- and diglycerides rich in unsaturated fat (ES Food Cor., Gunpo, Republic of Korea) were purchased from local markets.

### 2.2. Ice Cream Manufacturing

**Figure 1:**
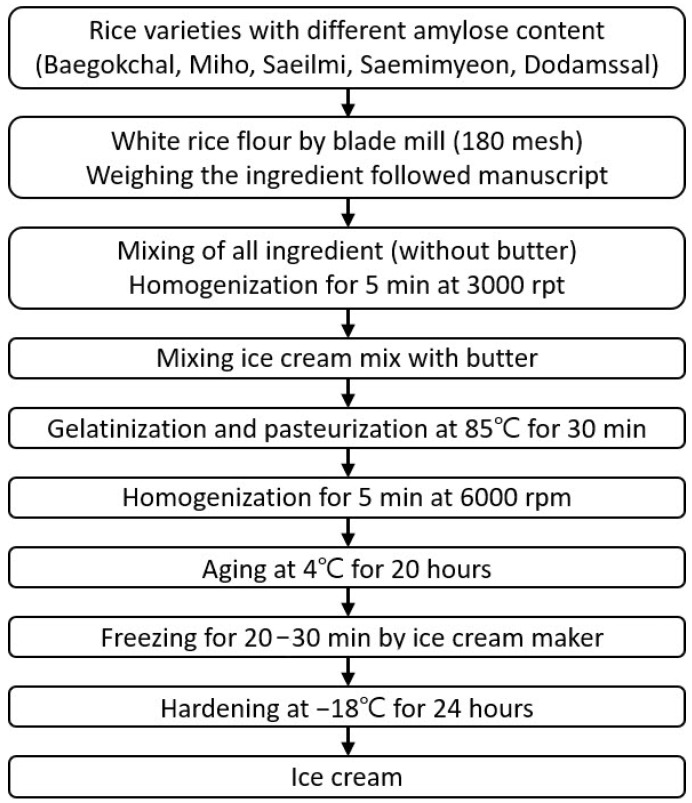
Schematic process flow diagram of ice cream preparation.

The manufacturing process of rice ice cream is shown in Figure 1. Rice flours with five different amylose contents (Baegokchal, Miho, Saeilmi, Saemimyeon, and Dodamssal) comprised 10% of the ingredients by weight, while skim milk powder comprised 4.5%, sugar 10%, glycerin esters of fatty acids 0.3%, locust bean gum 0.3%, and water 65%. First, these ingredients were weighed to prepare an ice cream mix with a weight of 600 g. Then, all ingredients were mixed at 3000 rpm for 5 min using a homogenizer (AM-9, Nihonseiki Kashima Co., Ltd., Tokyo, Japan). Thereafter, butter (adding 10% to the weight) was added to the ice cream mix. The ice cream mix was gelatinized and pasteurized at 85 °C for 30 min, and then homogenized at 6000 rpm for 5 min. The ice cream mix was placed in a glass bottle, aged at 4 °C for 20 h, and then was churned in an ice cream maker (DWI-5200, Macdous, Bucheon, Republic of Korea) operated in the strong mode for 20–30 min to manufacture rice ice cream. The ice cream was placed in a plastic container and hardened in a freezer at −18 °C for 24 h. This manufacturing process was repeated three times for each rice variety and used in subsequent experiments.

### 2.3. Characteristics of Processed Rice

#### 2.3.1. Amylose Content

Rice amylose content was determined using the methods of Juliano [7], according to solubilization and color development. Rice flour (0.1 g), ethanol (1 mL), and 1 N sodium hydroxide (9 mL) were added and incubated for 20 min at 90 °C, and the mixture was increased to a volume of 100 mL using distilled water. An aliquot of 5 mL was mixed with 1 N acetic acid (1 mL) and iodine solution (I_2_ in 2% KI, 2 mL), the volume was increased to 100 mL with distilled water, and the mixture was incubated for 20 min at 30 °C. Absorbance was measured at 620 nm using an ultraviolet-visible spectrometer (UV-2700, Shimadzu Co., Kyoto, Japan). The absorbance at 620 nm and the amylose concentrations were adjusted with a standard curve based on potato (*Solanum tuberosum* L.) starch [37]. 

#### 2.3.2. Crude Protein Content

Rice crude protein content was determined using near-infrared (NIR) spectroscopy (XM-1100 series, FOSS NIR Systems INC., Laurel, MD, USA). Rice flours (0.6 g) were added to the micro insert ring bound to the mini sample cup, the air gap in the sample was removed using the sample cup disposable back, and NIR spectroscopy was performed using visible and near-infrared wavelengths (400–2500 nm) under ambient temperature conditions (25 °C). The software ISI scan (version 4.5.0, InfraSoft International, Port Matilda, PA, USA) was used for the calculation of crude protein (R^2^ = 0.982) [38].

#### 2.3.3. Pasting Properties

Rice moisture content was measured using a moisture meter (MS-70, AND, Tokyo, Japan), and pasting properties were analyzed using a rapid viscosity analyzer (RVA 4500, Perten Instruments, Häqersten, Sweden). Rice flour (3.0 g, 12% moisture content) was added to an aluminum container for rapid viscosity analysis and 25 mL of distilled water was added. The samples were incubated at 50 °C for 1 min, then heated to 95 °C for 3.45 min, maintained at 95 °C for 2.7 min, cooled to 50 °C for 3.91 min, and maintained at 50 °C for 1.24 min. The parameters recorded were initial pasting temperature, peak viscosity (P), trough viscosity (T), final viscosity (F), breakdown viscosity (P − T), and setback viscosity (F − T) [39].

### 2.4. Physicochemical Properties of Rice Ice Cream

#### 2.4.1. Total Solids, Total Sugars, pH, Viscosity, and Hardness

Total solids, total sugars, pH, viscosity, and hardness of the rice ice creams were measured. The total solids were determined by measuring the moisture content using the air oven method. Rice ice cream (3 g) was transferred to a pre-weighed flat bottom aluminum dish, which was moved to a hot air oven set to 105 °C for 5 h. Dried rice ice cream was then placed in desiccators containing silica gel as a desiccant. After 30 min, the dish was weighed. Total solids were calculated by employing the following formula.

Total solid (%) = (W_3_ − W_1_)/(W_2_ − W_1_) × 100

W_1_: Weight of empty dish

W_2_: Weight of empty dish and rice ice cream

W_3_: Weight of empty dish and dried rice ice cream

Total sugars were measured using a hand refractometer (Master-α, ATAGO Co., Tokyo, Japan), and pH was measured using a pH meter (Orion 3 Star, Thermo Electron Co., Waltham, MA, USA).

The viscosity of rice ice cream was measured using a viscometer (DV1M, Brook Field Engineering, MA, USA) with a spindle (LV-04/64) after aging at 4 °C [40]. All measurements were recorded after 1 min at 20 rpm and reported as the apparent viscosity.

Hardness was measured using a rheometer (Compac-100II, SunScientific Co., Tokyo, Japan) after hardening the rice ice cream in a cube frame (24 mm × 24 mm × 24 mm) and aging it for 5 min at ambient temperature (25 °C) [26]. The hardness analysis of the ice cream samples was conducted using a rheometer with a 50 mm stainless steel cylinder probe. The speed was 30.0 mm min^−1^, the distance was 3.0 mm, and the cell load was 19.6133 N (Newton). The hardness was the peak value of the first compression.

#### 2.4.2. Overrun and Drip-Through Rate

Overrun is the increase in the volume of ice cream that occurs due to the incorporation of air during batch freezing. The method of measuring overrun involves separately weighing the ice cream mix and frozen ice cream in an affixed volume container (approximately 175 mL), then calculating the percentage increase in volume using the formula described by Akbari, Eskandari, Niakosari, and Bedeltavana [41]. The overrun measurement was carried out in triplicate for every other sample throughout the ice cream production process.

Overrun (%) = (W_1_ − W_2_)/W_2_ × 100

W_1_: Weight of ice cream mix

W_2_: Weight of ice cream

The rate at which ice cream melted through a mesh screen at room temperature was measured using the procedures specified by Bolliger et al. [42]. At ambient temperature (25 °C), 90 g of rice ice cream was quickly cut from the center of each sample and placed on the center of a metal screen (6 mm pore size mesh) resting above a beaker on a scale. The weight of the melted rice ice cream that had dripped through the screen was recorded every 10 min for 120 min. The weight of the dripped through portion (grams) was plotted against time (min). The slope of the linear portion of the curve was used to determine the drip-through rate (g/min). For each batch, the drip-through rate was recorded for every other sample throughout the ice cream production. The drip-through rate measurement was carried out in triplicate.

#### 2.4.3. Color

Color was measured using a colorimeter (CR-400, Konica Minolta, Tokyo, Japan). The parameters evaluated from the CIELAB system were lightness (L*), red/green (a*), and yellow/blue (b*). Rice ice cream was added to the measurement cell (50 × 15 mm), so that there was no empty space, and then hardened, and each sample was measured 10 times.

### 2.5. Sensory Evaluation

The ice creams were stored at −18 °C for over 24 h after production and remained at ambient temperature (25 °C ± 1 °C) for 5 min before sensory analysis [43]. A 25 g serving of ice cream was assessed by thirty panelists, previously trained in descriptive analysis methods [44], who performed a sensory evaluation using a modified method described by Chung, et al. [45]. The panelists were composed of graduate students or academics in the Food Science and Technology Department at Kyungpook National University (KNU). The sensory evaluation was conducted in an independent and controlled environment at room temperature. The panelists were instructed to rinse their mouths with distilled water between each sample to reduce any carryover effects. To prevent any bias, the order of presentation of the samples was randomized. The panelists evaluated the samples for color and appearance, taste, texture, chewy texture, aroma, rice flavor, and overall acceptance on a scale ranging from 1 (extremely weak or dislike) to 7 (extremely strong or like). All panelists signed an informed consent form before participating in the study. Sensory evaluation approval for the sensory study was obtained from the Institutional Review Board (IRB) at Kyungpook National University (IRB No. KNU-2021-0154).

### 2.6. Statistical Analysis

Values are expressed as the mean ± standard deviation (*n* = 3), and color measurement carried out 10 times. The results were evaluated by the analysis of variance and Duncan’s multiple range test (*p* < 0.05) using SAS (version 9.4, Cary, NC, USA). Correlation analysis was used to determine relationships among variables. The correlation between rice (amylose and crude protein, pasting properties) and rice ice cream (total solids, total sugars, pH, viscosity, hardness, overrun, drip-through rate, and color), using physicochemical properties, was determined using the Pearson correlation coefficient.

## 3. Results and Discussion

### 3.1. Characteristics of Processed Rice

The amylose content, crude protein, and pasting properties of rice are presented in Table 1. Amylose and crude protein content are critical factors that influence not only the taste and cooking, but also the physical properties of rice [46]. The average amylose content of Korean rice varieties is 19.99%, and the average protein content is 7.23% [47]. The rice amylose content used for rice ice cream production ranged from 5.89% to 40.39%, and the amylose content was ranked from lowest to highest for the Baegokchal (5.89%), Miho (12.40%), Saeilmi (17.40%), Saemimyeon (25.78%), and Dodamssal (40.39%) varieties (*p* < 0.05).

Rice grains contain a substantial amount of storage protein that can be classified as either prolamin, glutelin, albumin, or globulin [48]. Additionally, rice endosperm proteins, which constitute the majority of rice grain proteins, are mostly insoluble in water due to the high molecular weight of the rice glutelin profile [49]. The crude protein content of rice ranged from 6.44% to 6.90%, in the following order: Saeilmi (6.44%), Miho (6.69%), Baegokchal (6.71%), Saemimyeon (6.86%), and Dodamssal (6.90%). In a study by Park et al. [50], the crude protein content of domestically grown rice varieties Baegokchal, Ilmi, Mimyeon, and Dodamssal ranged from 5.66% to 7.17%, which is similar to the findings of this study.

The initial pasting temperature of rice ranged from 67.15 °C to 84.14 °C, the peak viscosity from 147.03 to 403.11 cP, the trough viscosity from 64.08 to 204.89 cP, the final viscosity from 82.50 to 386.41 cP, the breakdown viscosity from 99.33 to 205.33 cP, and the setback viscosity from 18.41 to 181.53 cP. As the amylose content increased, the initial pasting temperature, peak viscosity, trough viscosity, final viscosity, and setback viscosity of Baegokchal, Miho, Saeilmi, and Saemimyeon (but not Dodamssal) showed a positive relationship. According to research, such as that of Hasjim, et al. [51], it has been reported that the particle size and grinding method of rice flour can affect the pasting properties. Therefore, in this experiment, to reduce the variables that could affect the rice physicochemical properties and ice cream properties, each rice variety was ground in the same way and only samples that passed through 180 mesh (about particle size; <80 μm) were used for analysis and production [52]. A study by Suzuki et al. [53] found that the initial pasting temperature, trough viscosity, final viscosity, and setback viscosity are positively correlated with amylose content in the degree of polymerization of Japonica glutinous rice. Moreover, in the study by Balet et al. [54], amylose content significantly correlated with nearly all the pasting properties, except for peak viscosity, which was found to be greatly influenced by the environment. Specifically, the initial gelatinization temperature increased with increasing amylose content. This is because the starch particles of high amylose varieties are more densely packed than those of low amylose varieties, which increases resistance to swelling during gelatinization [55,56]. This is due to interactions between long chains or large crystal structures [57]. Among the pasting properties of Saemimyeon, peak viscosity (403.11 cP), trough viscosity (204.89 cP), final viscosity (386.41 cP), and setback viscosity (181.53 cP) were the highest among the varieties (*p* < 0.05). Saeilmi showed the highest breakdown viscosity, followed by Saemimyeon, Miho, Baegokchal, and Dodamssal (*p* < 0.05). These results clearly distinguish the rice characteristics and act as an important variable in the quality characteristics of rice ice cream manufactured using rice with different amylose contents.

### 3.2. Physicochemical Properties of Processed Rice Ice Cream

The physicochemical properties of rice ice cream, including total solids, total sugars, pH, viscosity, hardness, overrun, and drip-through rate, are shown in Table 2. The rice ice cream had a total solids content ranging from 33.06% to 33.65%, a total sugars content of 19.00 to 26.33 °Bx, pH values of 6.66 to 6.81, viscosity ranging from 2170 to 25,030 cP, hardness ranging from 4.27 to 49.55 Ncm^−2^, overrun ranging from 17.95% to 46.99%, and drip-through rate ranging from 0.75 to 0.98 g/min.

Erkaya, et al. [58] found that the total solids content of ice cream increases with the amount of raw materials used, making it an indicator of the raw materials. Therefore, by analyzing the range of total solids in our study, it can be confirmed that the ice cream was prepared using the same manufacturing process.

The total sugar content decreased as the amylose content increased (*p* < 0.05). This correlation can be attributed to the varying total sugars content produced during the gelatinization of each type of rice at 85 for 30 min. Varavinit et al. [59] found a positive correlation between amylose content and the initial pasting temperature of pasting properties. Additionally, a higher initial temperature was shown to result in a more difficult gelatinization progression under identical temperature conditions [60]. Therefore, the variation in total sugar content observed in our study demonstrated this difference in gelatinization progress.

**Figure 2:**
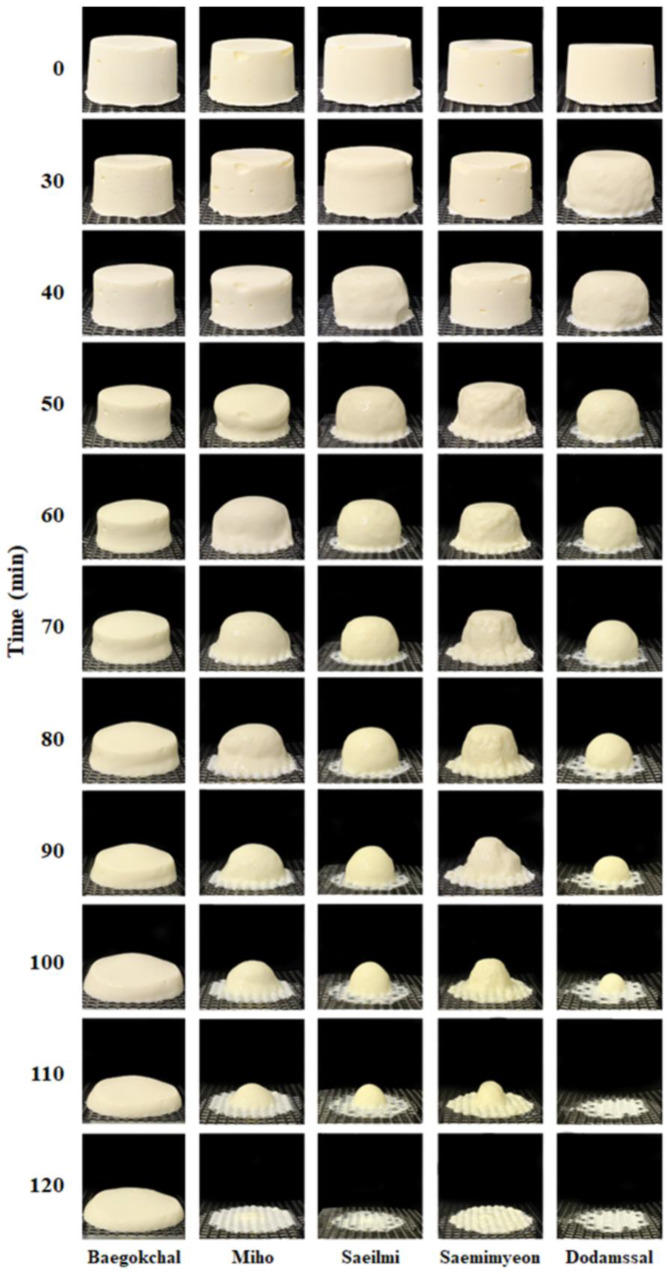
Photo of ice cream during the meltdown test.

Among the raw material grains for rice ice cream production, when prepared using Baegokchal glutinous rice, total solids content was 33.65%, total sugars content was 26.33 °Bx, pH was 6.81, and viscosity was 25,030 cP, which was the highest among the rice ice creams. The higher the amylose content, the lower the total solids, total sugars, pH, and viscosity (*p* < 0.05). The hardness of the rice ice cream was highest when prepared using Saemimyeon (49.55 Ncm^−2^), but the hardness of the rice ice cream prepared using Baegokchal was 4.27 Ncm^−2^, a difference of 11-fold, depending on the type of rice used. However, the Baegokchal overrun value was 46.99%, which was more than double that of Saemimyeon (17.95%). As such, hardness is affected by overrun, as well as rheological properties of the mixture [61], and hardness and overrun are inversely proportional to the current results. The overrun of ice cream is the percentage increase in volume that occurs when air is incorporated into the mix during freezing. Moreover, the overrun varies from 30% to 100%, in the case of general ice cream, whereas gelato ice cream, which is known worldwide for its sticky properties, is known to have little or no overrun [62]. Ice cream with a high overrun has a light taste and flavor and uses fewer ingredients, whereas ice cream with a low overrun has a relatively rich taste and flavor and contains more ingredients, so it is also called premium ice cream [63]. The drip-through rate was highest when using Dodamssal and Saeilmi, at 0.98 g/min, and decreased in the order of Miho (0.89 g/min) and Saemimyeon (0.75 g/min) (*p* < 0.05). Baegokchal did not pass through the mesh until the 120 min mark, when all other samples were melted and were clustered on the top of the mesh (Figure 2). This is thought to be due to the high content of solids added to the ice cream, as well as its high viscosity, helping the ice cream maintain its shape while it slowly melts [64].

**Figure 3:**
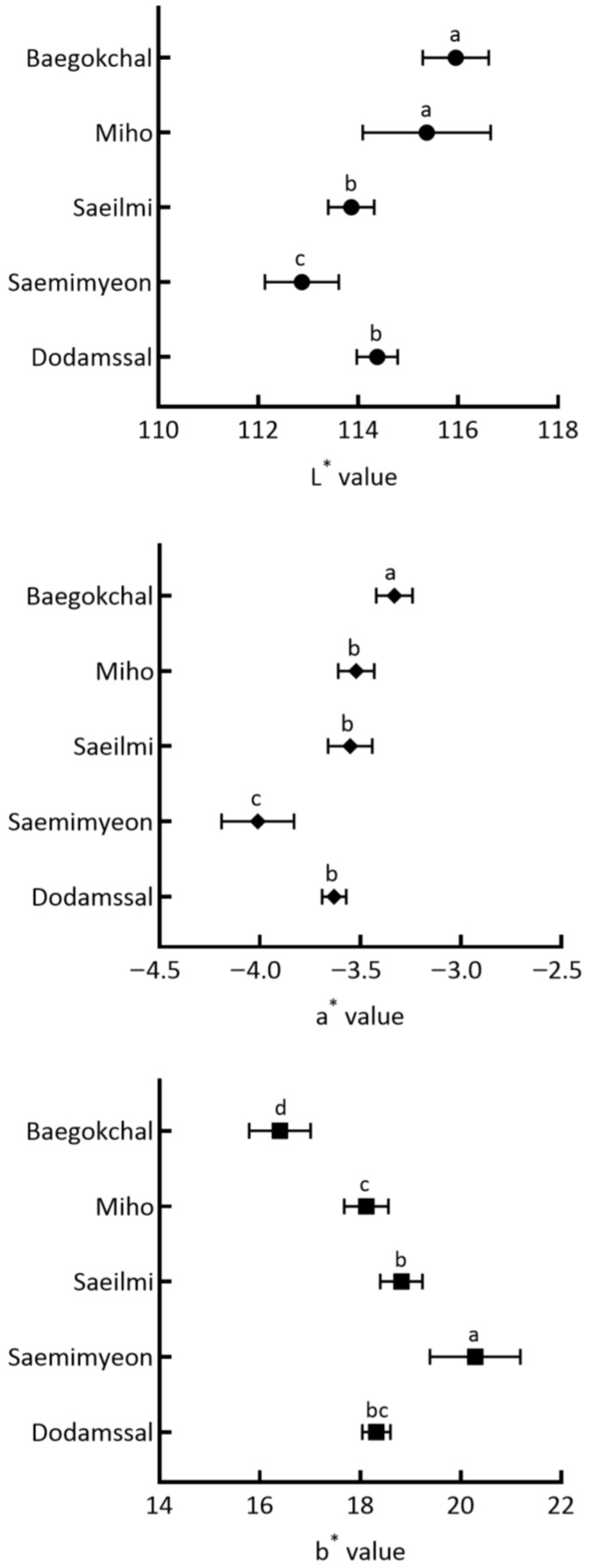
Color value of ice cream made with processed rice. Means with different letters above a bar are significantly different at *p* < 0.05.

The color values of rice ice cream are shown in Figure 3. Color value is an important quality characteristic that affects consumer preference, as it determines the appearance quality of ice cream [65]. These colors are usually preferred because they are reminiscent of the traditional appearance of rice and the flavor and aroma of rice ice cream. Moreover, the whiter the raw processed grain rice, the higher its value in the market [66]. The L* value, which indicates brightness, ranged from 112.87 to 115.95, with rice ice cream prepared using Baegokchal and Miho with low amylose content having high L* values of 115.95 and 115.37, respectively. Therefore, Baegokchal and Miho are anticipated to have the highest preference value for ice cream, based on color. However, the L* value of rice ice cream using Saemimyeon, a high amylose rice, was the lowest at 112.87 (*p* < 0.05). According to Lamberts et al. [67], the brightness value is between 85–100, depending on the milling degree and rice variety, and the homogenized rice powder scatters light in the visible spectrum region, thus showing a high brightness value overall. In addition, rice with a high amylose content is less water-absorbent, resulting in harder and brighter rice grains, whereas rice with a low amylose content is more water-absorbent, resulting in softer and more transparent rice grains [68]. For this reason, there may be a difference in L* values when using rice with different amylose contents.

The degree of redness (a* value), representing red to green, was weakly green, with a negative value ranging from −4.01 to −3.33 for all of the rice ice creams. The rice ice cream prepared using Baegokchal had the highest a* value of −3.33, while the lowest was the ice cream made with Saemimyeon at −4.01 (*p* < 0.05). On the other hand, the b* value, which ranged from yellow to blue, was yellow, with a positive value ranging from 16.40 to 20.29 for all of the rice ice creams. Saemimyeon had the highest b* value of 20.29, whereas Baegokchal had the lowest at 16.40 (*p* < 0.05). Based on these results, the L* and a* values decreased, while the b* value increased as the amylose content of the processed rice used for the production of rice ice cream increased.

### 3.3. Correlation between Processed Rice and Rice Ice Cream Quality Characteristics

**Figure 4:**
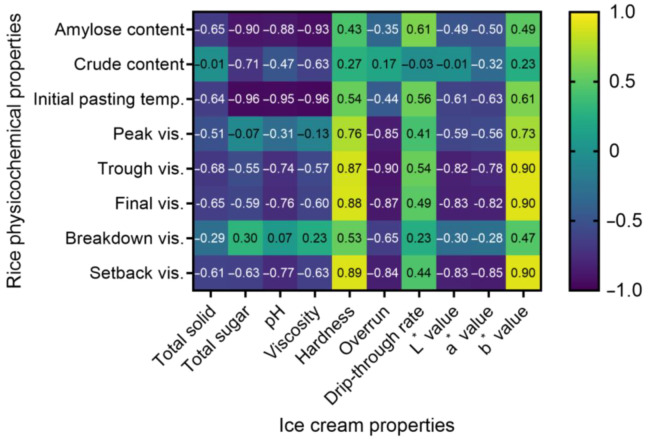
Correlation coefficients heat map between physicochemical properties of ice cream and characteristics of processed rice. Yellow indicates a positive association, and blue indicates a negative association.

The relationship between the processed rice (amylose content, crude protein content, and pasting properties) and the physicochemical properties of rice ice cream (total solids, total sugars, pH, viscosity, hardness, overrun, drip-through rate, and color value) are shown in Figure 4. The total solids content of rice ice cream showed a high negative correlation with rice trough viscosity (r = −0.683, *p* < 0.01). The total sugars content, pH, and viscosity of the rice ice cream showed the highest negative correlation with initial pasting temperature at −0.956, −0.947, and −0.963, respectively, while the amylose content also showed a high negative correlation with these variables at −0.897, −0.877, and −0.932, respectively. Rice ice cream hardness showed the highest correlation with setback viscosity (r = 0.894), whereas overrun showed a negative correlation with trough viscosity (r = −0.895). The setback value is an indicator of the retrogradation of rice starch, which occurs in the third stage of the series of RVA tests. Retrogradation refers to the process in which starch molecules break the initial gelation and reform a gel network by interacting with water molecules [69]. This process changes the molecular structure of the starch and affects the hardness and texture of the final product. According to Yoenyongbuddhagal and Noomhorm [70], the setback value in the pasting properties results of rice flour highly correlated with the hardness of the processed product. Therefore, a higher setback value in rice flour indicates that retrogradation has increased in the flour after processing, which may lead to a harder and tougher texture in the final product.

When analyzing the correlation between the color value of rice ice cream and rice physicochemical properties, the lightness value (L*) of rice ice cream showed the highest negative correlation with final viscosity (r = −0.834, *p* < 0.001), and also showed a high negative correlation with setback viscosity (r = −0.827) and trough viscosity (r = −0.824). The redness value (a*) showed the highest negative correlation with setback viscosity (r = −0.851, *p* < 0.001), and also showed a high negative correlation with final viscosity (r = −0.823). The yellowness value (b*) showed the highest positive correlation with final viscosity (r = 0.904, *p* < 0.001), while trough viscosity and setback viscosity showed a very high positive correlation (r = 0.902, *p* < 0.001). Setback and final viscosity are closely related to the amylose content [71]. According to the results of Ahmed et al. [72], if the setback and final viscosity are high, a significant amount of retrogradation has occurred, resulting in a firm and stable gel. Thus, it can be concluded that starch retrogradation has worked well in this case. It has been reported that as the density and stiffness of the rice structure increase, the color index decreases [73,74].

Thus, the total sugars, pH, viscosity, and drip-through rate among the quality characteristics of rice ice cream were affected by the amylose content and initial pasting temperature, while the color value was affected by rice final viscosity and setback viscosity.

### 3.4. Sensory Evaluation of Rice Ice Cream

The sensory evaluation results of rice ice cream are presented in Table 3. The study evaluated several attributes, including color, appearance, taste, texture, chewy texture, aroma, rice flavor, and overall preference. The color and appearance ratings ranged from 5.3 to 5.7, indicating a moderate to high preference among the samples. Similarly, the aroma scores ranged from 4.6 to 5.2, indicating a generally positive evaluation of the ice cream’s smell.

However, the samples exhibited notable differences in terms of texture, chewy texture, and rice flavor (*p* < 0.05). Among the samples, Miho and Saeilmi scored the highest in terms of texture preference (5.5 and 5.4, respectively), while Dodamssal received the lowest score (3.9). The chewy texture was also evaluated, and the results indicated that Baegokchal scored the highest (6.2), followed by Miho, Saeilmi, Saemimyeon, and Dodamssal, with significant differences between the samples (F-value = 9.68). These findings emphasize the importance of texture in determining the overall acceptance of rice ice cream.

The overall preference scores ranged from 4.1 to 5.1, indicating that the participants generally liked the rice ice cream samples. Interestingly, there was little difference in color preference among the rice varieties (F-value = 0.21), indicating that the choice of rice did not significantly affect the color of the ice cream. A higher F-value indicates a higher proportion of the variance explained by the model, which suggests that the corresponding factor has a significant overall effect. Thus, the F-values for texture and chewy texture were high, indicating that these attributes had a more important impact on the overall evaluation. In fact, previous research has shown that texture is the most important factor influencing consumer preferences when choosing ice cream, followed by taste and aroma [75]. Therefore, if the chewy texture of rice ice cream is too strong or weak, it can affect the overall acceptance, even if the aroma and rice flavor are good.

In conclusion, based on the sensory evaluation results, it is suggested that texture may be considered a more important attribute for determining the overall acceptance of rice ice cream. Therefore, the processing quality characteristics of rice ice cream should be carefully considered and adjusted based on the rice variety to meet consumer needs and preferences.

## 4. Conclusions

In this study, rice ice cream was produced by incorporating processed rice with different amylose contents and comparing the resulting quality characteristics. The physicochemical properties of ice cream, including overrun and drip-through rate, were measured and compared among the different rice varieties. Significant differences were observed in the pH, total sugars content, and total solids of the ice cream with different amylose contents. The correlation between the physicochemical properties of rice and ice cream was shown, based on the different properties of rice ice cream. The correlation between the quality properties of rice and rice ice cream showed that the setback and final viscosity were highly correlated with hardness and color value. By utilizing the relationship between rice varieties and processing quality characteristics, this data can be used for the production of rice ice cream that meets consumer needs.

This preliminary investigation provides some useful information regarding the suitability of employing rice varieties with different amylose contents as alternative raw materials in ice cream making with regard to the nutraceutical, nutritional, and physiochemical properties. This may help promote rice processing and create a new market for rice production.

## Figures and Tables

**Table 1:** Amylose, crude protein, and pasting properties of processed rice.

Variety	Amylose Content (%)	Crude Protein (%)	Pasting Properties					
			Initial Pasting Temp. (°C)	Peak Vis. (cP)	Trough Vis. (cP)	Final Vis. (cP)	Breakdown vis. (cP)	Setback Vis. (cP)
Baegokchal	5.89 ± 0.04 ^e1^	6.71 ± 0.02 ^b^	67.15 ± 0.52 ^e^	163.42 ± 1.80 ^d^	64.08 ± 0.44 ^e^	82.50 ± 0.80 ^e^	99.33 ± 1.72 ^c^	18.41 ± 0.55 ^e^
Miho	12.40 ± 0.21 ^d^	6.69 ± 0.05 ^b^	69.12 ± 0.06 ^d^	295.42 ± 6.41 ^c^	100.25 ± 2.72 ^d^	150.50 ± 3.29 ^d^	195.16 ± 4.19 ^b^	50.25 ± 2.08 ^d^
Saeilmi	17.40 ± 0.37 ^c^	6.44 ± 0.06 ^c^	73.47 ± 0.56 ^c^	374.89 ± 2.05 ^b^	169.56 ± 5.10 ^b^	277.86 ± 7.02 ^b^	205.33 ± 3.36 ^a^	108.30 ± 1.93 ^b^
Saemimyeon	25.78 ± 0.85 ^b^	6.86 ± 0.07 ^a^	80.28 ± 0.03 ^b^	403.11 ± 3.72 ^a^	204.89 ± 2.68 ^a^	386.41 ± 1.89 ^a^	198.22 ± 1.87 ^b^	181.53 ± 1.28 ^a^
Dodamssal	40.39 ± 0.22 ^a^	6.90 ± 0.05 ^a^	84.12 ± 0.45 ^a^	147.03 ± 0.67 ^e^	114.14 ± 0.84 ^c^	181.75 ± 2.63 ^c^	32.89 ± 1.21 ^d^	67.61 ± 3.01 ^c^

cP, centipoise; vis, viscosity; temp, temperature. Amylose content (%) is reported on an ‘as is’ basis. Crude protein (%) is reported on an ‘as is’ basis. ^1^ Mean ± standard deviation (*n* = 3) within each column followed by different letters is significantly different (*p* < 0.05).

**Table 2:** Physicochemical properties of ice cream.

Variety	Total Solids (%)	Total Sugars (°Bx)	pH	Viscosity (cP)	Hardness (Ncm^−2^)	Overrun (%)	Drip-Through Rate (g/min)
Baegokchal	33.65 ± 0.07 ^a1^	26.33 ± 0.58 ^a^	6.81 ± 0.01 ^a^	25,030 ± 1813 ^a^	4.27 ± 2.37 ^c^	46.99 ± 3.17 ^a^	-
Miho	33.14 ± 0.02 ^b^	25.67 ± 0.58 ^b^	6.77 ± 0.01 ^b^	17,920 ± 1763 ^b^	27.87 ± 4.30 ^b^	27.77 ± 1.80 ^c^	0.89 ± 0.03 ^ab^
Saeilmi	33.06 ± 0.01 ^c^	25.00 ± 0.00 ^c^	6.73 ± 0.01 ^c^	17,180 ± 711 ^b^	27.22 ± 0.61 ^b^	20.19 ± 2.26 ^d^	0.98 ± 0.01 ^a^
Saemimyeon	33.10 ± 0.01 ^bc^	19.00 ± 0.00 ^d^	6.66 ± 0.01 ^d^	4030 ± 142 ^c^	49.88 ± 9.52 ^a^	17.95 ± 1.83 ^d^	0.75 ± 0.17 ^b^
Dodamssal	33.07 ± 0.03 ^c^	19.00 ± 0.00 ^d^	6.67 ± 0.01 ^d^	2170 ± 154 ^c^	23.54 ± 0.61 ^b^	32.06 ± 1.11 ^b^	0.98 ± 0.00 ^a^

Total solids (%) are reported on an ‘as is’ basis. ^1^ Mean ± standard deviation (*n* = 3) within each column followed by different letters is significantly different (*p* < 0.05).

**Table 3:** Sensory scores on a 7-point numeric scale for ice cream made with processed rice.

Variety	Color and Appearance	Taste	Texture	Chewy Texture	Aroma	Rice Flavor	Overall Acceptance
Baegokchal	5.7 ± 0.7 ^a1^	3.7 ± 1.2 ^a^	4.8 ± 1.2 ^ab^	6.2 ± 1.1 ^a^	5.2 ± 1.3 ^a^	5.3 ± 1.1 ^a^	4.1 ± 1.8 ^a^
Miho	5.7 ± 1.1 ^a^	4.6 ± 1.3 ^a^	5.5 ± 0.8 ^a^	5.0 ± 0.0 ^b^	5.1 ± 1.6 ^a^	3.7 ± 1.4 ^b^	5.1 ± 0.6 ^a^
Saeilmi	5.5 ± 1.3 ^a^	4.6 ± 1.5 ^a^	5.4 ± 1.1 ^a^	4.7 ± 0.9 ^b^	4.8 ± 1.7 ^a^	4.0 ± 1.2 ^ab^	5.1 ± 1.3 ^a^
Saemimyeon	5.6 ± 1.1 ^a^	4.7 ± 1.4 ^a^	4.8 ± 1.3 ^ab^	3.2 ± 1.4 ^c^	4.7 ± 1.6 ^a^	4.8 ± 1.5 ^ab^	4.9 ± 1.7 ^a^
Dodamssal	5.3 ± 1.3 ^a^	4.4 ± 1.3 ^a^	3.9 ± 1.8 ^b^	2.5 ± 1.1 ^c^	4.6 ± 1.8 ^a^	4.7 ± 1.8 ^ab^	4.5 ± 1.6 ^a^
F-value	0.21	0.91	2.46	9.68 ***^2^	0.25	2.03	0.64

Sensory scores were assessed on a 7-point scale, where 1 = extremely weak or dislike, and 7 = extremely strong or like. ^1^ Mean ± standard deviation (*n* = 3) within each column followed by different letters is significantly different (*p* < 0.05). ^2^ *** significant at *p* < 0.001.

## Data Availability

The data presented in this study are available in the article or supplementary material.

## Supplementary Materials

The following supporting information can be downloaded at: https://www.mdpi.com/article/10.3390/foods12071518/s1, Figure S1: Distribution of amylose content of rice varieties; Table S1: Molecular genetics and general amylose content of processed rice; Table S2: Proximate composition of processed rice; Table S3: Mineral content of processed rice.
