# Supplementary material for: Quality Characteristics of Rice-Based Ice Creams with Different Amylose Contents
Source: Foods. 2023 Apr 3;12(7):1518. doi: 10.3390/foods12071518 (PMC10094488; doi:10.3390/foods12071518)
Supplement: Supplementary file 1 [file foods-12-01518-s001.zip › foods-2277872-supplementary.pdf]

Supplementary Materials

Figure S1. Distribution of amylose content of rice varieties

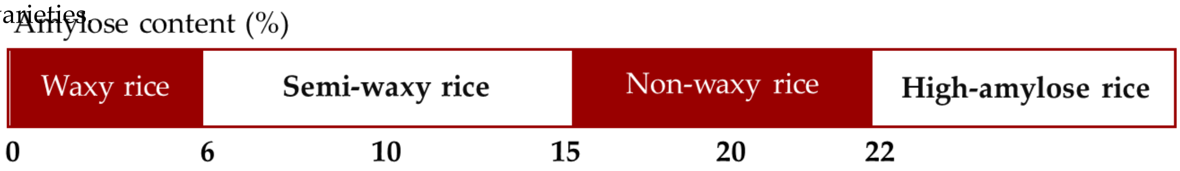

Table S1. Molecular genetics and general amylose content of processed rice.

Variety	Type	Cross	Marker name	Traits
Baegokchal	Japonica	Dongjinchal//YR17334Acp24/Hwayeong	wx	Waxy
Miho	Japonica	Iksan480/Milky Princess	wx-mq	Semi-waxy
Saeilmi	Japonica	Ilmi*5/Hwayeong	Wx	Non-waxy
Saemimyeon	Indica (Tongil-type)	Milyang181//IR50/YR22791	Wx^a/Wx^b	High-amylose
Dodamssal	Japonica	Goami/Goami2	GBSS1+SBE3	High-amylose + Resistant starch

Table S2. Proximate composition of processed rice.

Variety	Moisture (%)	Crude protein (g/100 g)	Crude fat (g/100 g)	Carbohydrate (g/100 g)	Crude ash (%)
Baegokchal	7.34	6.84	3.07	82.29	0.46
Miho	6.99	6.99	2.62	83.01	0.39
Saeilmi	7.00	6.27	2.72	83.58	0.42
Saemimyeon	8.56	7.55	2.35	81.29	0.25
Dodamssal	10.68	7.06	3.73	78.03	0.50

Table S3. Mineral content of processed rice.

(mass%)

Variety	Zn	Ca	P	K	Mg	Al	Si	S	Cl
Baegokchal	0.00±0.00[a][1]	0.01±0.00[a]	0.15±0.00[b]	0.22±0.00a	0.14±0.01[a]	0.01±0.00[a]	0.01±0.00[a]	0.08±0.00[d]	0.02±0.00[c]
Miho	0.00±0.00[a]	0.01±0.00[a]	0.13±0.00[c]	0.17±0.00d	0.12±0.00[b]	0.01±0.00[a]	0.01±0.00[a]	0.09±0.00[a]	0.02±0.00[c]
Saeilmi	0.00±0.00[a]	0.01±0.00[a]	0.10±0.00[d]	0.15±0.00e	0.09±0.00[c]	0.01±0.00[a]	0.01±0.00[a]	0.08±0.00[b]	0.03±0.00[a]
Saemimyeon	0.00±0.00[a]	0.01±0.00[a]	0.15±0.00[b]	0.18±0.00c	0.13±0.00[a]	0.01±0.00[a]	0.01±0.00[a]	0.09±0.00[a]	0.03±0.00[b]
Dodamssal	0.00±0.00[a]	0.01±0.00[a]	0.15±0.00[a]	0.20±0.00[b]	0.14±0.00[a]	0.01±0.00[a]	0.01±0.00[a]	0.08±0.00[c]	0.02±0.00[d]
**Variety**	**Fe**	**Cu**	**Mn**	**Ge**	**Se**	**Pb**	**Na**	**Ag**	**Au**
Baegokchal	0.00±0.00[a]	0.00±0.00[a]	0.00±0.00[a]	ND[2]	ND	ND	ND	ND	ND
Miho	0.00±0.00[a]	0.00±0.00[a]	0.00±0.00[a]	ND	ND	ND	ND	ND	ND
Saeilmi	0.00±0.00[a]	0.00±0.00[a]	0.00±0.00[a]	ND	ND	ND	ND	ND	ND
Saemimyeon	0.00±0.00[a]	0.00±0.00[a]	0.00±0.00[a]	ND	ND	ND	ND	ND	ND
Dodamssal	0.00±0.00[a]	0.00±0.00[a]	0.00±0.00[a]	ND	ND	ND	ND	ND	ND

[1] Mean ± standard deviation (n=3) within each column followed by different letters are significantly different (p<0.05).

[2] ND; not detected.
